# Rhizospheric microbiomics integrated with plant transcriptomics provides insight into the Cd response mechanisms of the newly identified Cd accumulator *Dahlia pinnata*

**DOI:** 10.3389/fpls.2022.1091056

**Published:** 2022-12-15

**Authors:** Xiong Li, Boqun Li, Tao Jin, Huafang Chen, Gaojuan Zhao, Xiangshi Qin, Yongping Yang, Jianchu Xu

**Affiliations:** ^1^ Department of Economic Plants and Biotechnology, Yunnan Key Laboratory for Wild Plant Resources, Kunming Institute of Botany, Chinese Academy of Sciences, Kunming, China; ^2^ Center for Mountain Futures, Kunming Institute of Botany, Chinese Academy of Sciences, Kunming, China; ^3^ Germplasm Bank of Wild Species, Kunming Institute of Botany, Chinese Academy of Sciences, Kunming, China; ^4^ Science and Technology Information Center, Kunming Institute of Botany, Chinese Academy of Sciences, Kunming, China; ^5^ Xishuangbanna Tropical Botanical Garden, Chinese Academy of Sciences, Xishuangbanna, China

**Keywords:** heavy metal contamination, phytoextraction, *Dahlia pinnata*, rhizobacteria, signal transduction

## Abstract

Phytoremediation that depends on excellent plant resources and effective enhancing measures is important for remediating heavy metal-contaminated soils. This study investigated the cadmium (Cd) tolerance and accumulation characteristics of *Dahlia pinnata* Cav. to evaluate its Cd phytoremediation potential. Testing in soils spiked with 5–45 mg kg^–1^ Cd showed that *D. pinnata* has a strong Cd tolerance capacity and appreciable shoot Cd bioconcentration factors (0.80–1.32) and translocation factors (0.81–1.59), indicating that *D. pinnata* can be defined as a Cd accumulator. In the rhizosphere, Cd stress (45 mg kg^–1^ Cd) did not change the soil physicochemical properties but influenced the bacterial community composition compared to control conditions. Notably, the increased abundance of the bacterial phylum Patescibacteria and the dominance of several Cd-tolerant plant growth–promoting rhizobacteria (e.g., *Sphingomonas*, *Gemmatimonas*, *Bryobacter*, *Flavisolibacter*, *Nocardioides*, and *Bradyrhizobium*) likely facilitated Cd tolerance and accumulation in *D. pinnata*. Comparative transcriptomic analysis showed that Cd significantly induced (*P* < 0.001) the expression of genes involved in lignin synthesis in *D. pinnata* roots and leaves, which are likely to fix Cd^2+^ to the cell wall and inhibit Cd entry into the cytoplasm. Moreover, Cd induced a sophisticated signal transduction network that initiated detoxification processes in roots as well as ethylene synthesis from methionine metabolism to regulate Cd responses in leaves. This study suggests that *D. pinnata* can be potentially used for phytoextraction and improves our understanding of Cd-response mechanisms in plants from rhizospheric and molecular perspectives.

## 1 Introduction

Cadmium (Cd) is a toxic soil contaminant worldwide (Fan et al., 2022) and preventing it from entering the human body from the environment *via* the food chain (Mao et al., 2022) is a great challenge. Many techniques have been developed to minimize Cd absorption and accumulation in crops (Rizwan et al., 2016; Liu et al., 2018; Shi et al., 2020), but such measures can only temporarily address the problem. The long-term solution is to remove Cd from contaminated soils.

Phytoextraction is a sustainable remediation strategy that removes soil Cd by harvesting the aboveground parts of Cd (hyper)accumulators (Oladoye et al., 2022). Although many potential Cd (hyper)accumulators have been screened from naturally or artificially Cd-contaminated soils (Reeves et al., 2018), their widespread application is restricted by insufficient biomass accumulation, Cd bearing capacity, and environmental adaptability (Shen et al., 2021). Thus, further screening and molecular-assisted breeding are needed to identify or produce ideal Cd phytoextractors. Alternatively, uncovering the mechanisms of rhizospheric effects (Khanna et al., 2019) on plant responses to Cd will also benefit the development of feasible strategies that enhance phytoextraction efficiency.

The largest angiosperm family, Asteraceae, has served as an important source of Cd (hyper)accumulators (Nikolic and Stevovic, 2015; Reeves et al., 2018). *Dahlia pinnata* Cav., a perennial herb of Asteraceae with a large biomass (**Supplementary Figure S1**), is one of the most widely cultivated ornamental plants worldwide (Liu et al., 2010). The wide cultivation of this species implies its strong adaptability to diverse environments. These characteristics indicate its natural advantages in the phytoremediation of heavy metal-contaminated soils. To date, the accumulation characteristics of chromium, lead, and arsenic in *D. pinnata* have been explored (Ramana et al., 2013; Cui et al., 2014; Raza et al., 2019), but few data are available on the Cd tolerance and accumulation of this species.

In this study, we analyze the Cd tolerance and accumulation capacity of *D. pinnata* while determining how its rhizospheric microenvironments (especially rhizobacteria) varied under Cd stress. Additionally, we performed transcriptomic analysis to explore the molecular underpinnings of the Cd stress response in *D. pinnata*. This study will improve our understanding of the Cd response mechanisms in plants from rhizospheric and molecular perspectives.

## 2 Materials and methods

### 2.1 Experimental design

#### 2.1.1 Pot experiments

The soils used for a previous study (Li X. et al., 2022) were sieved and mixed to obtain homogeneous composite soil. The Cd concentration gradients of 0 (Cd0), 5 (Cd5), 20 (Cd20), and 45 (Cd45) mg Cd kg^−1^ dry soil, which were chosen according to several previous studies (Wu et al., 2018; Dhaliwal et al., 2020), were set to understand whether *D. pinnata* can be defined as a Cd hyperaccumulator. The weighed Cd (via CdCl_2_•2.5H_2_O) was dissolved in an appropriate amount of deionized water, and the solutions were adequately mixed with the Cd-free soils to obtain soils with targeted Cd concentrations. Actual Cd concentrations (Cd5: 6.84 mg kg^−1^, Cd20: 20.10 mg kg^−1^, and Cd45: 45.40 mg kg^−1^) were measured prior to experiments. The prepared soils were equally loaded into uniform flowerpots (Li X. et al., 2022) with impermeable plastic trays to catch leachates. The soils were equilibrated for 1 month in a glass greenhouse (light: approximately 82% natural light, 12–14 h, 23–25°C; darkness: 10–12 h, 18–20°C; humidity: 40–60%) at Kunming Institute of Botany, Chinese Academy of Sciences.

Mature *D*. *pinnata* seeds purchased from a horticultural company (Wuhan, China) were surface-sterilized, cleaned, and sown (three seeds per pot) in the aforementioned flowerpots. One seedling was left in each pot after sprouting. Plants were watered regularly, and pots were position-changed to control for microenvironment variation. After 3 months of growth, plant roots, stems, and leaves, as well as rhizospheric soils, were collected following published methods (Wu et al., 2018; Li X. et al., 2022). Cd^2+^ adsorbed on the root surface was removed using Na_2_EDTA solution (15 mM, 20 min) (Wu et al., 2018). Three biological replicates were prepared.

#### 2.1.2 Hydroponic experiments

To explore the molecular mechanisms of the Cd response without interference from soil, *D. pinnata* was grown hydroponically. Seeds were surface-sterilized (Li et al., 2021b) and germinated in culture dishes (25 ± 2°C, 14 h light/10 h dark). Two weeks later, seedlings in similar size were individually cultured in 50 mL plastic tubes containing 1/2 modified Hoagland nutrient solution (Zhou et al., 2016) for 15 d in the above-mentioned greenhouse. The culture medium was replaced every 3 d. Subsequently, the plants were transferred into two hydroponic boxes (l × w × h: 37.5 cm × 25.0 cm × 13.5 cm; 12 seedlings per box) with 10 L 1/2 modified Hoagland nutrient solution (renewed every 3 d). One month later, seedlings in one box were treated with 50 μM Cd^2+^ (via CdCl_2_•2.5H_2_O), an appropriate Cd concentration that can effectively trigger changes in plant gene expression in a short time (Chen et al., 2021; Wang et al., 2022), whereas those in the other remained untreated as controls.

After 48 h of treatment, two to three top leaves and all roots of each plant were harvested separately. For transcriptomic analysis, samples were immediately frozen in liquid nitrogen and stored at −80°C. For Cd analysis, samples were cleaned for Cd^2+^ removal using Na_2_EDTA solution (15 mM, 20 min) (Wu et al., 2018) and oven-dried at 80°C for 48 h. Three biological replicates were prepared for each measurement.

### 2.2 Measurement of Cd concentration

Cd concentrations in dried *D. pinnata* samples were measured following previously described methods (Li et al., 2017). Bioconcentration factors (BCFs), translocation factors (TFs), and Cd content in shoots and roots were calculated using published formulas (Li et al., 2021a).

### 2.3 Determination of soil physicochemical properties

Published methods (Li et al., 2021a; Li X. et al., 2022; Yang et al., 2022) were employed to measure the following variables in rhizospheric soils of *D. pinnata*: pH, cation exchange capacity (CEC), hydrolyzable nitrogen (HN), available phosphorus (AP), available potassium (AK), total Cd (TCd), and available Cd (ACd).

### 2.4 Soil bacterial community analysis

DNA extraction, 16S rDNA amplification, sequencing, and bioinformatics analyses were performed as previously described (Li et al., 2021a), with some modifications. In brief, soil microbial DNA was extracted using the HiPure Soil DNA Kit (Magen, Guangzhou, China), and the V3–V4 region of the 16S rDNA gene was amplified by PCR for Illumina NovaSeq 6000 sequencing using the primer pair 341F (5′−CCTACGGGNGGCWGCAG−3′) and 806R (5′−GGACTACHVGGGTATCTAAT−3′). The raw sequencing data were filtered to obtain clean reads, which were further merged and filtered to obtain effective tags. Thereafter, the effective tags were clustered into operational taxonomic units (OTUs). The representative sequences in each cluster were classified into organisms (confidence threshold value: 0.8) based on the SILVA database (version 132) (Pruesse et al., 2007). Alpha diversity analysis was conducted using QIIME (version 1.9.1) (Caporaso et al., 2010). Venn analysis between groups was performed in the R project VennDiagram package (version 1.6.16) (Chen and Boutros, 2011) to identify unique and common species and OTUs. Species comparison between groups was calculated by Welch’s *t*-test in the R project Vegan package (version 2.5.3) (Dixon, 2003).

### 2.5 *De novo* transcriptomic analysis

Published methods were followed for RNA extraction and sequencing (Li et al., 2021b). Sequencing employed qualified libraries and the BGI high-throughput platform DNBSEQ-T7 (BGI, Shenzhen, China). Bioinformatics analysis of sequencing results followed previous methods (Li et al., 2021b). Briefly, the number of reads aligned to each unigene was obtained in RSEM (Li and Dewey, 2011), and the results were converted to fragments per kilobase per million bases. Next, differential expression analysis was performed in DESeq2 (Love et al., 2014). Differentially expressed genes (DEGs) were those with expression fold change > 2 and *P* < 0.05. Functional analyses (Gene Ontology [GO] terms and KEGG pathways) of DEGs were performed using the OmicShare platform (http://www.omicshare.com/tools).

Ten DEGs in *D. pinnata* roots and leaves were randomly selected for validation with quantitative real-time PCR (qRT‐PCR), as previously described (Li et al., 2021b). Primer pairs and PCR product sizes are provided in **Supplementary Table S1**. The glyceraldehyde-3-phosphate dehydrogenase gene (Yuan et al., 2012) was used as the internal control.

### 2.6 Data analysis

Between-group differences were determined using one-way analysis of variance with Tukey’s test (n ≥ 3) or independent-sample *t* test (n = 2). Principal component analysis (PCA) was generated in OmicShare (http://www.omicshare.com/tools).

## 3 Results

### 3.1 Cd tolerance and accumulation characteristics of *D. pinnata*

**Figure 1:**
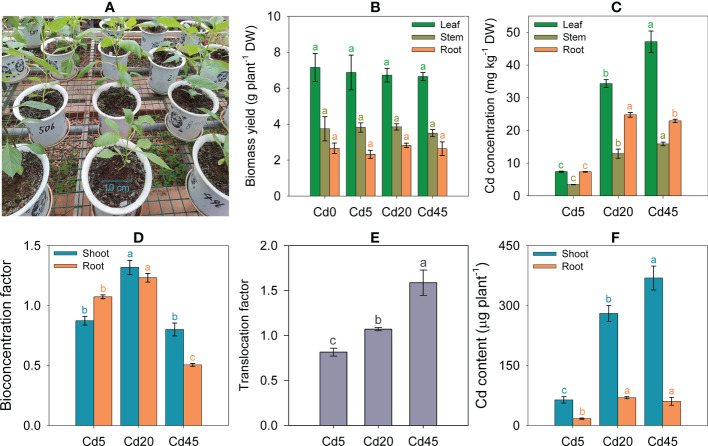
Cd tolerance and accumulation characteristics of *Dahlia pinnata*. **(A)** Greenhouse pot experiments growing *D pinnata* in soils spiked with 0 (Cd0), 5 (Cd5), 20 (Cd20), and 45 mg kg^–1^ Cd (Cd45). Biomass yields **(B)**, Cd concentrations **(C)**, bioconcentration factors **(D)**, translocation factors **(E)**, and Cd content **(F)** in *D. pinnata* under different treatments. Data represent means ± standard deviations (**B–F**: n = 3). Bars of the same color labeled with different letters indicate significant differences (*P* < 0.05, analysis of variance and Tukey’s test) between groups. DW: dry weight.

None of the Cd treatment (5–45 mg kg^–1^) caused visible toxic symptoms in *D. pinnata* (**Figure 1A**). Additionally, biomass yields of shoots and roots remained stable after 3 months regardless of Cd concentrations (**Figure 1B**). Thus, *D. pinnata* appears tolerant to Cd.

Cd concentrations in *D. pinnata* tissues increased significantly (*P* < 0.05) with increasing soil Cd concentrations. Under identical soil Cd concentrations, tissue Cd concentrations were highest in leaves, followed by roots and stems (**Figure 1C**). The average Cd concentrations in shoots were 5.98, 26.51, and 36.35 mg kg^–1^ under Cd5, Cd20, and Cd45, respectively (**Supplementary Figure S2**). The average shoot Cd BCFs ranged from 0.80 to 1.32 under different soil Cd concentrations, whereas the root Cd BCFs ranged from 0.51 to 1.23 (**Figure 1D**). The Cd20 condition yielded the highest Cd BCF in both shoots and roots (**Figure 1D**). The average Cd TFs ranged from 0.81 to 1.59, increasing (*P* < 0.05) as the soil Cd concentrations increased (**Figure 1E**). Approximately 78.9–86.2% of the total Cd accumulated in shoots under different soil Cd concentrations (**Figure 1F**).

### 3.2 Changes in the physicochemical indices of *D. pinnata* rhizosphere soils

The rhizospheric microenvironment exhibited large differences in TCd and ACd concentrations between Cd0 and Cd45 soils (**Supplementary Table S2**). However, the two soils had similar pH, CEC, HN, AP, and AK concentrations (**Supplementary Table S2**).

### 3.3 Dynamics of bacterial communities in the rhizosphere of *D. pinnata*

#### 3.3.1 Richness, diversity, and composition of bacterial communities

Raw reads from our high-throughput sequencing of the rhizospheric bacterial community were deposited in the NCBI Sequence Read Archive (SRA) (accession number: PRJNA874226). The samples generated 117,148–134,460 raw paired-end sequencing reads and 2,129–2,427 bacterial OTUs (**Supplementary Table S3**).

**Figure 2:**
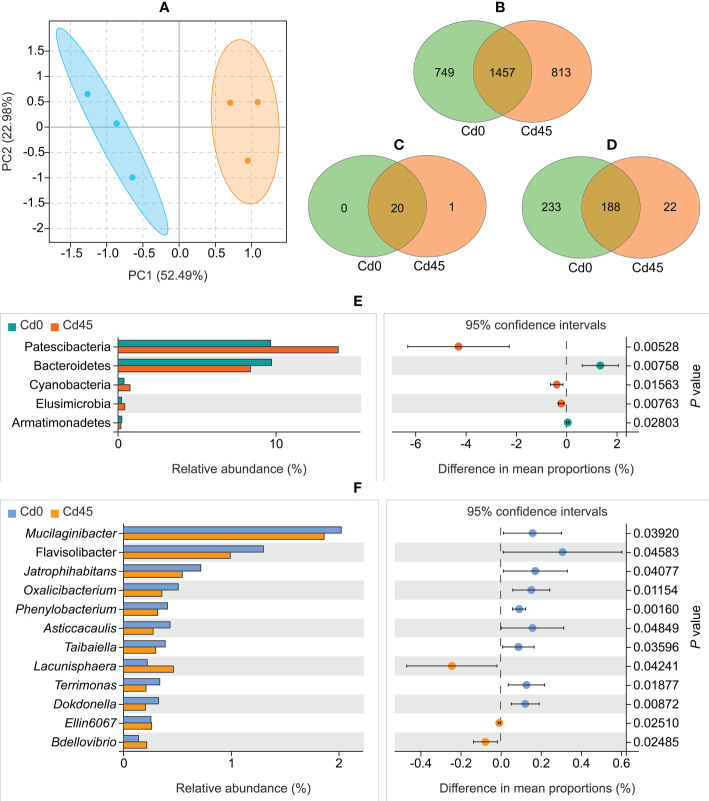
Rhizospheric bacterial community composition of *Dahlia pinnata* grown in soils spiked with 0 (Cd0) and 45 mg kg^–1^ Cd (Cd45). **(A)** Principal component analysis (PCA) of operational taxonomic units (OTUs). Venn diagram of bacterial OTUs **(B)**, phyla **(C)**, and genera **(D)** between Cd0 and Cd45 soils. Relative abundance of bacterial phyla **(E)** and genera **(F)** that differ between Cd0 and Cd45 soils. *P* < 0.05, significant using Welch’s *t*-test.

The PCA showed that Cd0- and Cd45-treated samples formed distinct clusters (**Figure 2A**). However, bacterial communities did not differ significantly in four alpha indices (Shannon, Simpson, Chao1, and Ace) between Cd0 and Cd45 soils (**Supplementary Table S4**). The two soils shared 1,457 core bacterial OTUs (**Figure 2B**), accounting for 66.0% and 64.2% of the total OTUs in the Cd0 (2,206) and Cd45 (2,270) soils, respectively. We identified 21 bacterial phyla and 233 genera, with most of them (20 phyla and 188 genera) common across the Cd0 and Cd45 soils (**Figures 2C, D**). Additionally, both soils had at least one sample that contained 16 bacterial phyla (**Supplementary Table S5**) and 75 bacterial genera (**Supplementary Table S6**) with a relative abundance of > 0.1%.

#### 3.3.2 Variations in microbial taxa between Cd0 and Cd45 soils

The Cd0 and Cd45 soils differed significantly in microbial taxa composition (*P* < 0.05, Welch’s *t*-test). The abundance of the phyla Patescibacteria, Cyanobacteria, and Elusimicrobia increased in the Cd45 soil compared with that in the Cd0 soil, whereas the abundance of the phyla Bacteroidetes and Armatimonadetes decreased (**Figure 2E**). Additionally, 12 bacterial genera differed in abundance between Cd0 and Cd45 soils (**Figure 2F**).

### 3.4 Comparative transcriptomics in *D. pinnata* roots and leaves

#### 3.4.1 Sequencing and quantitative results

**Figure 3:**
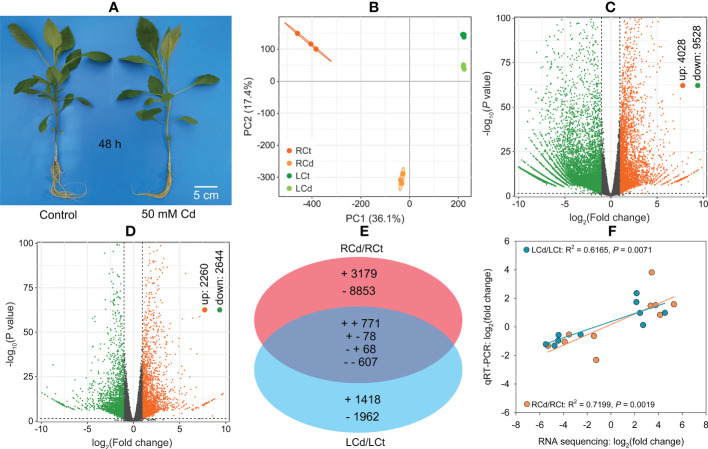
Transcriptomic analysis and qRT‐PCR validation for *Dahlia pinnata* grown in control and Cd treatment (50 mM, 48 h) conditions. **(A)** Morphology of *D. pinnata* after experimental treatment under hydroponic conditions. **(B)** Principal component analysis (PCA) based on unigene abundance. **(C)** Volcano plot of differentially expressed genes (DEGs) between control and Cd-treated roots. **(D)** Volcano plot of DEGs between control and Cd-treated leaves. **(E)** Venn diagram showing DEG variation between roots and leaves. **(F)** Linear regression of gene expression changes between RNA sequencing and qRT‐PCR for 10 selected genes in roots and leaves. RCt, control group of root; RCd, Cd-treated group of root; LCt, control group of leaf; LCd, Cd-treated group of leaf.

After 50 mM Cd treatment for 48 h under hydroponic conditions (**Figure 3A**), root and leaf Cd concentrations reached 11,900 and 227 mg kg^−1^, respectively (**Supplementary Figure S3**).

Approximately 67,988,424–68,000,000 raw RNA reads, which were deposited in the NCBI SRA (accession number: PRJNA811758), were generated for 12 samples, with clean reads accounting for 99% (**Supplementary Table S7**). High Q20 (> 97%) and Q30 (> 92%) values (**Supplementary Table S7**) indicated good sequencing quality. Of the 219,444 unigenes obtained, 91,692 (41.8%) were annotated in different databases (**Supplementary Table S8**). We quantified 40,679 (**Supplementary Table S9**) and 29,304 unigenes (**Supplementary Table S10**) in *D. pinnata* roots and leaves, respectively.

The PCA results (**Figure 3B**) indicated that Cd stress had a greater influence on gene expression in roots than in leaves. Under Cd stress, *D. pinnata* roots and leaves had 13,556 (4,028 upregulated and 9,528 downregulated) and 4,904 DEGs (2,260 upregulated and 2,644 downregulated), respectively (**Figures 3C, D**; **Supplementary Tables S9** and **S10**). We observed tissue-specific expression for 88.8% (roots) and 68.9% (leaves) of DEGs (**Figure 3E**). The remaining 1,524 DEGs were identified in both roots and leaves, and 90.4% exhibited the same change patterns (**Figure 3E**).

#### 3.4.2 qRT‐PCR validation

Significant correlations between the RNA sequencing and qRT‐PCR data in both roots (R^2^ = 0.7199, *P* < 0.01) and leaves (R^2^ = 0.6165, *P* < 0.01) (**Figure 3F**) were observed, indicating reliable RNA sequencing results in this study.

#### 3.4.3 GO analysis

In both roots and leaves, DEGs were enriched in similar GO terms under the biological process, molecular function, and cellular component categories (**Supplementary Figures S4** and **S5**). Nearly all enriched terms contained upregulated and downregulated genes (**Supplementary Figures S4** and **S5**), suggesting that Cd stress exerts multiple physiological effects on *D. pinnata*.

#### 3.4.4 KEGG pathway enrichment

**Figure 4:**
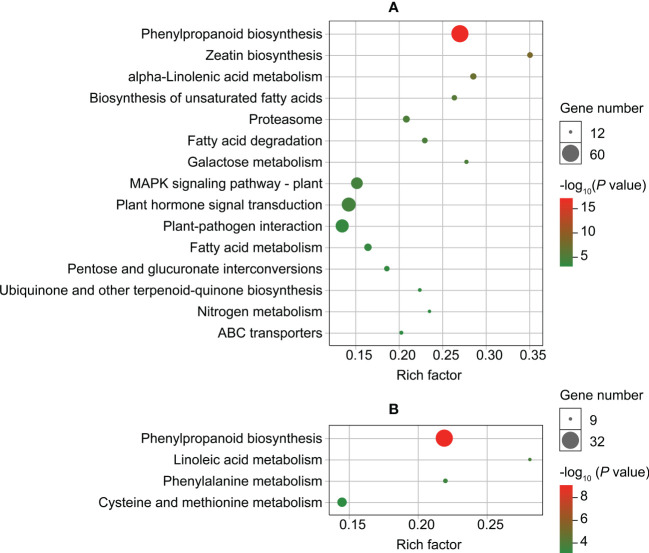
Enriched KEGG pathways (*P* < 0.001) of up-regulated genes in roots **(A)** and leaves **(B)** of *Dahlia pinnata* grown in control and Cd treatment (50 mM, 48 h) conditions.

The results of enrichment analysis on upregulated genes found that they were significantly enriched (*P* < 0.001) in 15 KEGG pathways in roots and four in leaves (**Figure 4**; **Supplementary Table S11**). The pathways were primarily associated with substance metabolism, signal transduction, and substance transport (**Figure 4**). Only one pathway (phenylpropanoid biosynthesis) was enriched in both roots and leaves (**Figure 4**).

We selected several pathways for further analysis based on the number of enriched genes (**Figure 4**). For roots, the pathways were related to phenylpropanoid biosynthesis, mitogen-activated protein kinase (MAPK) signaling, plant hormone signal transduction, and plant‐pathogen interaction; for leaves, pathways were involved in phenylpropanoid biosynthesis and cysteine/methionine metabolism. Many upregulated genes were involved in key reactions of these pathways (**Supplementary Figures S6–S11**), providing insight into the mechanisms of Cd tolerance in *D. pinnata*.

## 4 Discussion

### 4.1 Cd phytoextraction potential of *D. pinnata*

Cd tolerance and accumulation characteristics can determine plant suitability for phytoremediation (Li X. et al., 2022). Here, our experiments demonstrated that *D. pinnata* has strong Cd tolerance based on morphology and biomass accumulation in Cd-contaminated soils (**Figures 1A, B**). Moreover, the evidence supports *D. pinnata* as a phytoremediator because it accumulated Cd as the soil Cd concentrations rose (**Figure 1C**). Its appreciable Cd BCFs and TFs (**Figures 1D, E**) were near the thresholds of Cd hyperaccumulators (Li J. T. et al., 2018). Other promising findings are the high Cd concentrations in *D. pinnata* after short-term Cd treatment under hydroponic conditions (**Supplementary Figure S3**). However, the Cd accumulation capacity of *D. pinnata* seems dependent on soil Cd concentrations, given the changes in Cd BCFs and TFs under different Cd treatments (**Figures 1D, E**). In particular, the Cd BCF began to decline at Cd45 (**Figure 1D**), suggesting that Cd uptake was suppressed. The Cd transport rates in *D. pinnata* plants may be provoked by increasing Cd stress (**Figure 1E**), which can be interpreted as a detoxification strategy in the roots of some plants (Xue et al., 2019). Shoot Cd concentrations, BCFs, and TFs of *D. pinnata* did not reach those of Cd hyperaccumulators (Li J. T. et al., 2018). Nevertheless, our results suggest that *D. pinnata* qualifies as a Cd accumulator (Baker, 1981).

The vast majority (78.9–86.2%) of Cd accumulated in the shoots (**Figure 1F**), suggesting that *D. pinnata* can potentially be used for phytoextraction in a suitable range of Cd-contaminated soils. The large biomass and substantial growth rate of *D. pinnata* should be a considerable advantage compared with those of many other Cd (hyper)accumulators (Shen et al., 2021) when used for phytoextraction. Many follow-up studies would be useful to promote the application of *D. pinnata* for Cd. For example, more efficient cultivars for phytoextraction can be screened and identified from large germplasm resources (Liu et al., 2010). Alternatively, some targeted measures can be explored to further enhance the Cd phytoextraction efficiency of *D. pinnata*.

### 4.2 Effects of rhizospheric microenvironments on *D. pinnata* Cd response

As the region where roots interact with soils (York et al., 2016), the rhizosphere is critical to Cd tolerance and accumulation in plants. In this study, we found that pH, CEC, and nutrients (i.e., HN, AP, and AK) were similar between the Cd0- and Cd45-treated rhizospheres of *D. pinnata* (**Supplementary Table S2**), suggesting a relatively stable nutrient supply for the normal growth of *D. pinnata* plants under Cd stress.

Rhizospheric microbes have a strong influence on plant responses to heavy metals in soils (Hakim et al., 2021). In this study, Cd stress did not alter the total richness or diversity of the rhizospheric bacterial community (**Supplementary Table S4**), which is likely attributable to the same reasons as previous studies: a dynamic equilibrium of bacterial taxa (Li et al., 2021a; Li X. et al., 2022). The stable abundance of the most dominant phylum Proteobacteria (**Supplementary Table S5**), which is important to soil C and N cycles (Narendrula-Kotha and Nkongolo, 2017), between the Cd0 and Cd45 soils is probably related to HN stability (**Supplementary Table S2**). The variation in abundance of other rhizospheric bacterial phyla (**Figure 2E**) illustrates a preference or sensitivity to Cd and other soil factors. Notably, the second most dominant phylum, Patescibacteria (**Supplementary Table S5**), has been found to increase Cd tolerance in *Photinifraseri frase* by regulating heat shock proteins (Liu et al., 2022), indicating that the upregulation of Patescibacteria (**Figure 2E**) may play a similar role in the Cd response of *D. pinnata*.

Some Cd accumulators recruit plant growth–promoting rhizobacteria (PGPR) to cope with Cd stress, implying PGPR abundance typically increases under Cd contamination (Wang et al., 2020; Li et al., 2021a; Li X. et al., 2022). In this study, we were unable to determine the exact functions of these bacterial genera with differential abundance between Cd0 and Cd45 soils (**Figure 2F**). However, we identified several important PGPR (Abhilash et al., 2016), including *Sphingomonas*, *Gemmatimonas*, *Bryobacter*, *Flavisolibacter*, *Nocardioides*, and *Bradyrhizobium*, among the top 10 abundant bacterial genera (**Supplementary Table S6**). All these PGPR except *Flavisolibacter* remained stable between Cd0 and Cd45 soils (**Figure 2F**; **Supplementary Table S6**). Their abundance in Cd45 soil may allow *D. pinnata* to resist Cd through multiple mechanisms (Khanna et al., 2019; Yang et al., 2022). Some of these PGPR can also regulate phytoextraction (Khanna et al., 2019; Yang et al., 2022), for instance, by affecting Cd solubility and ameliorating soil physicochemical conditions (Dary et al., 2010; Guo and Chi, 2014; Wang et al., 2019; Asaf et al., 2020). Notably, *Sphingomonas* can secrete phytochemicals as byproducts to enhance heavy metal (e.g., Cd, copper, zinc, and nickel) bioavailability (Asaf et al., 2020). Thus, the abundant *Sphingomonas* in the Cd45 soil likely contributes strongly to Cd uptake in *D. pinnata*.

### 4.3 Gene network in *D. pinnata* roots and leaves under Cd stress

Transcriptomics is useful for revealing the regulatory network of the Cd response in plants (Li et al., 2021b; Wang et al., 2021; Wang et al., 2022). In this study, we performed a hydroponic experiment to avoid the effects of other soil factors on our transcriptome analysis. After 48 h of Cd treatment, *D. pinnata* roots absorbed a high concentration of Cd, with a small portion transferred to the shoots (**Supplementary Figure S3**). This large difference in Cd concentrations is likely the cause of distinct DEGs between roots and leaves (**Figure 3E**). KEGG enrichment analysis was conducted using the upregulated genes to understand potential Cd tolerance mechanisms in *D. pinnata*. The results showed that various metabolic processes, signal transduction, or substance transport pathways in *D. pinnata* were significantly induced (*P* < 0.001) by Cd (**Figure 4**), suggesting their important roles in Cd tolerance in *D. pinnata*. In the following sections, we discuss major stress-response processes that shed light on Cd detoxification mechanisms.

#### 4.3.1 Phenylpropanoid biosynthesis in roots and leaves

**Figure 5:**
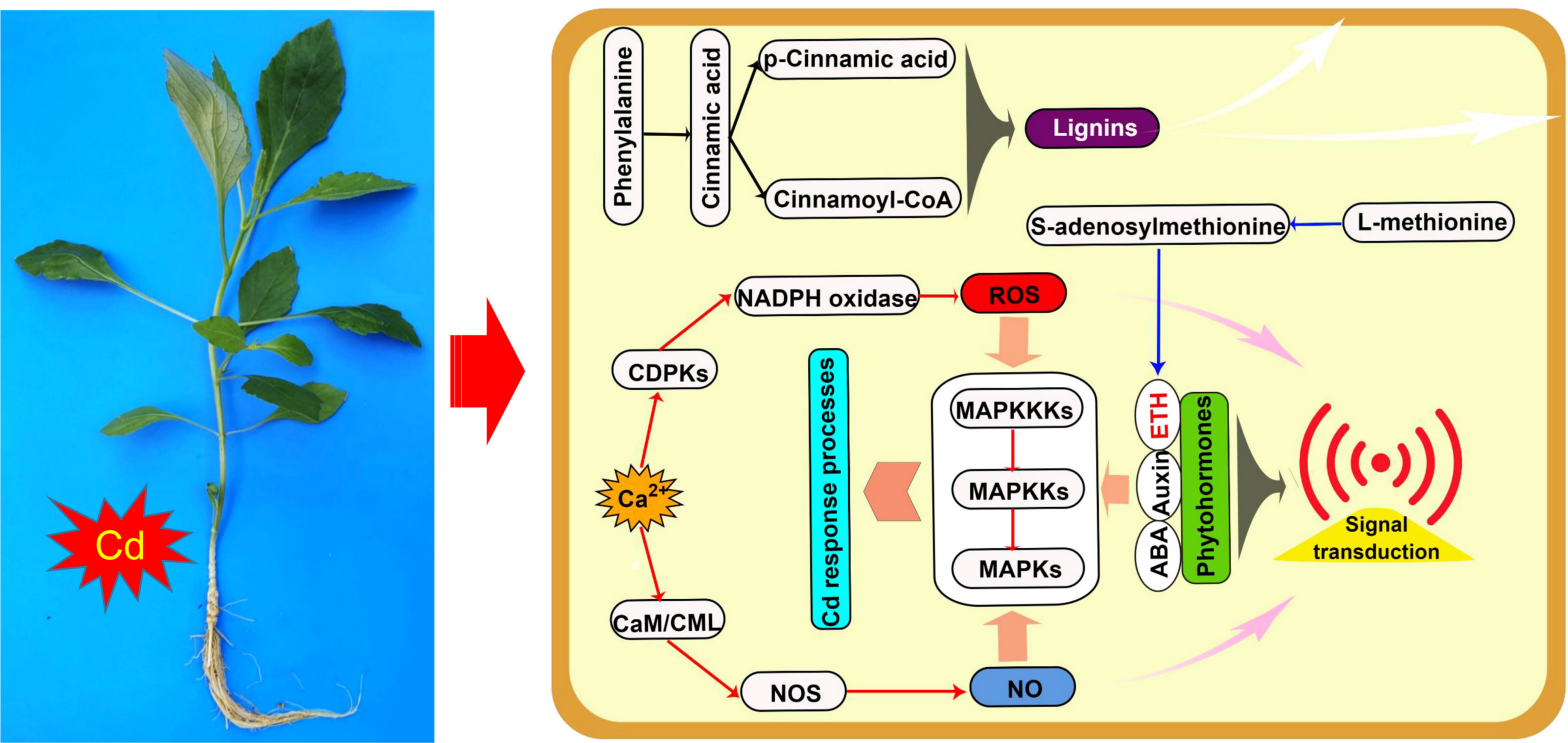
Schematic of Cd response processes of *Dahlia pinnata* grown in control and Cd treatment (50 mM, 48 h) conditions. Specific processes in roots and leaves are represented using red and blue arrows, respectively; common processes are represented using black arrows. ABA, abscisic acid; CaM/CML, calmodulin/calmodulin-like; CDPK, calcium-dependent protein kinase; ETH, ethylene; MAPK, mitogen-activated protein kinase; MAPKK, MAPK kinase; MAPKKK, MAPKK kinase; NO, nitric oxide; NOS, NO synthase.

The downstream products of the phenylpropanoid metabolism pathway, such as lignins, flavonoids, and procyanidins, improve plant tolerance to heavy metals through multiple mechanisms (Ge et al., 2022). In this study, upregulated genes were highly enriched in the synthesis process of lignins in the phenylpropanoid metabolism pathway in both the roots and leaves of *D. pinnata* (**Supplementary Figures S6** and **S10**), which may lead to an increase in the production of various lignins (e.g., syringyl lignin, 5-hydroxyguaiacyl lignin, and guaiacyl lignin). Lignins, as the main components of the secondary wall of plant cells, can fix heavy metal ions in the cell wall through their functional groups, such as carboxyl, phenolic, and aldehyde groups, to inhibit the entry of heavy metals into the cytoplasm (Li Y. et al., 2018; Su et al., 2020; Yu et al., 2023). Therefore, the results suggest that lignin-mediated cell wall compartmentalization (**Figure 5**) may be an important Cd detoxification mechanism in both the roots and leaves of *D. pinnata*.

#### 4.3.2 Signal transduction networks in roots

The ability of plants to perceive, transmit, and translate stress signals into an appropriate physiological response determines their ability to tolerate heavy metals (Islam et al., 2015). Plant responses are typically modulated *via* crosstalk between signaling molecules such as Ca^2+^, reactive oxygen species (ROS), nitric oxide (NO), phytohormones, and hydrogen sulfide (Luo et al., 2016). In this study, many upregulated genes in *D. pinnata* roots were enriched in three signaling pathways that are likely critical to Cd tolerance: MAPK signaling (**Supplementary Figure S7**), hormone signal transduction (**Supplementary Figure S8**), and plant‐pathogen interaction (**Supplementary Figure S9**).

As a second messenger, Ca^2+^ plays an important role in intracellular signaling pathways of acclimation to heavy metals and other stressors. Ca^2+^ enters the cytoplasm through cyclic nucleotide-gated calcium channels (CNGCs) and interacts with calcium-binding proteins, including calcium-dependent protein kinase (CDPK), calmodulin (CaM), and calmodulin-binding protein kinase (Li Y. Q. et al., 2022). Of these, CDPKs are involved in phosphorylating NADPH oxidase to produce ROS (Li Y. Q. et al., 2022), whereas the Ca^2+^/CaMs complex promotes NO synthase activity to produce NO (He et al., 2015). Here, we observed that Cd induced CNGC activity, along with Ca^2+^-related ROS and NO production, in *D. pinnata* roots (**Supplementary Figure S9**). These results strengthen the hypothesis that intracellular Ca^2+^, ROS, and NO are pivotal in plant acclimation to heavy-metal stress (Luo et al., 2016).

Phytohormones such as ethylene (ETH), abscisic acid (ABA), auxin, jasmonic acid, and salicylic acid often act as signals that trigger a plant’s heavy-metal stress response (Saini et al., 2021). Unsurprisingly, Cd treatment upregulated signaling pathways in *D. pinnata* roots that involve auxin, ABA, and ETH (**Supplementary Figure S9**), supporting the importance of phytohormone signal transduction in the Cd stress response.

Heavy metal exposure also initiates MAPK cascades in plants as part of signal transduction (Mondal, 2022). Consistent with previous research (Li S. C. et al., 2022; Mondal, 2022), ETH, ABA, and H_2_O_2_ were primarily responsible for initiating MAPK cascades in *D. pinnata* roots under Cd stress (**Supplementary Figure S7**). Our results support the idea of crosstalk between MAPK cascades and other signaling molecules during the coordination of *D. pinnata* responses to Cd stress.

In summary, Cd stress induced a complex signal transduction network involving Ca^2+^, ROS, NO, phytohormones, and MAPK cascades in *D. pinnata* roots (**Figure 5**). This network likely triggers detoxification processes that ensure *D. pinnata* tolerance to Cd.

#### 4.3.3 Methionine metabolism in leaves

As a source of sulfur, methionine is required for the biosynthesis of glutathione and phytochelatins, which contribute to heavy-metal detoxification in plants (Thakur et al., 2022). Methionine is also involved in metal uptake and transport in plants (Mousavi et al., 2021), as well as being the immediate precursor of *S*-adenosylmethionine, itself a precursor to ETH and polyamine biosynthesis. In this study, Cd stress upregulated genes encoding enzymes key to methionine synthesis of ETH in *D. pinnata* leaves (**Supplementary Figure S11**). Recent evidence suggests that ETH mediates Cd resistance in plants by positively regulating flavonoid biosynthesis and antioxidant activity (Chen et al., 2022). Moreover, in the Cd hyperaccumulator *Sedum alfredii*, high ETH concentrations postponed apoplastic-barrier formation and thus promoted Cd accumulation in root apoplasts (Liu et al., 2021). Taken together, the data strongly imply that ETH signaling based on methionine metabolism (**Figure 5**) is critical to regulating Cd stress responses in *D. pinnata* leaves.

## 5 Conclusions

This study identified *D. pinnata* as a Cd accumulator for phytoextraction based on its strong Cd tolerance capacity, as well as appreciable shoot BCFs and TFs in Cd-contaminated soils. In the *D. pinnata* rhizosphere, high Cd concentrations did not change the soil physicochemical properties but had an effect on the bacterial community composition. Notably, the increased abundance of Patescibacteria and the dominance of some Cd-tolerant PGPR likely facilitated Cd tolerance and accumulation in *D. pinnata*. Comparative transcriptomics showed that Cd significantly induced the expression of genes involved in lignin synthesis in *D. pinnata* roots and leaves. Moreover, Cd induced a sophisticated signal transduction network to initiate molecular and cellular detoxification processes in roots, whereas ethylene synthesis based on methionine metabolism likely regulates Cd responses in leaves. These results reveal the mechanism of the *D. pinnata* response to Cd stress. More research is needed to fully unlock the potential of *D. pinnata* in heavy metal phytoextraction. For example, the most suitable *D. pinnata* cultivars should be screened, and the actual Cd phytoextraction capacity in natural soils requires verification. Nevertheless, our study highlights a new Cd accumulator and provides a preliminary outline for understanding its Cd response mechanisms, with the resultant information beneficial for developing Cd phytoextraction enhancement measures.

## Data availability statement

The datasets presented in this study can be found in online repositories. The names of the repository/repositories and accession number(s) can be found below: https://www.ncbi.nlm.nih.gov/, PRJNA874226; https://www.ncbi.nlm.nih.gov/, PRJNA811758.

## Author contributions

XL: Conceptualization, Methodology, Investigation, Validation, Formal analysis, Writing - Original Draft, Visualization, Funding acquisition. BL: Investigation. TJ: Formal analysis, Writing - Review & Editing, Funding acquisition. HC: Formal analysis, Writing - Review and Editing. GZ: Formal analysis, Writing - Review and Editing. XQ: Investigation. YY: Writing - Review and Editing, Supervision. JX: Writing - Review & Editing, Supervision. All authors approved the final version of the manuscript.
