# Supplementary material for: Rhizospheric microbiomics integrated with plant transcriptomics provides insight into the Cd response mechanisms of the newly identified Cd accumulator Dahlia pinnata
Source: Front Plant Sci. 2022 Dec 15;13:1091056. doi: 10.3389/fpls.2022.1091056 (PMC9798219; doi:10.3389/fpls.2022.1091056)
Supplement: Supplementary file 1 [file Image_1.pdf]

Supplementary Material

Rhizospheric microbiomics integrated with plant transcriptomics provides insight into the Cd response mechanisms of the newly identified Cd accumulator *Dahlia pinnata*

Xiong Li*, Boqun Li, Tao Jin, Huafang Chen, Gaojuan Zhao, Xiangshi Qin, Yongping Yang, Jianchu Xu

*** Correspondence:** Xiong Li: lixiong@mail.kib.ac.cn

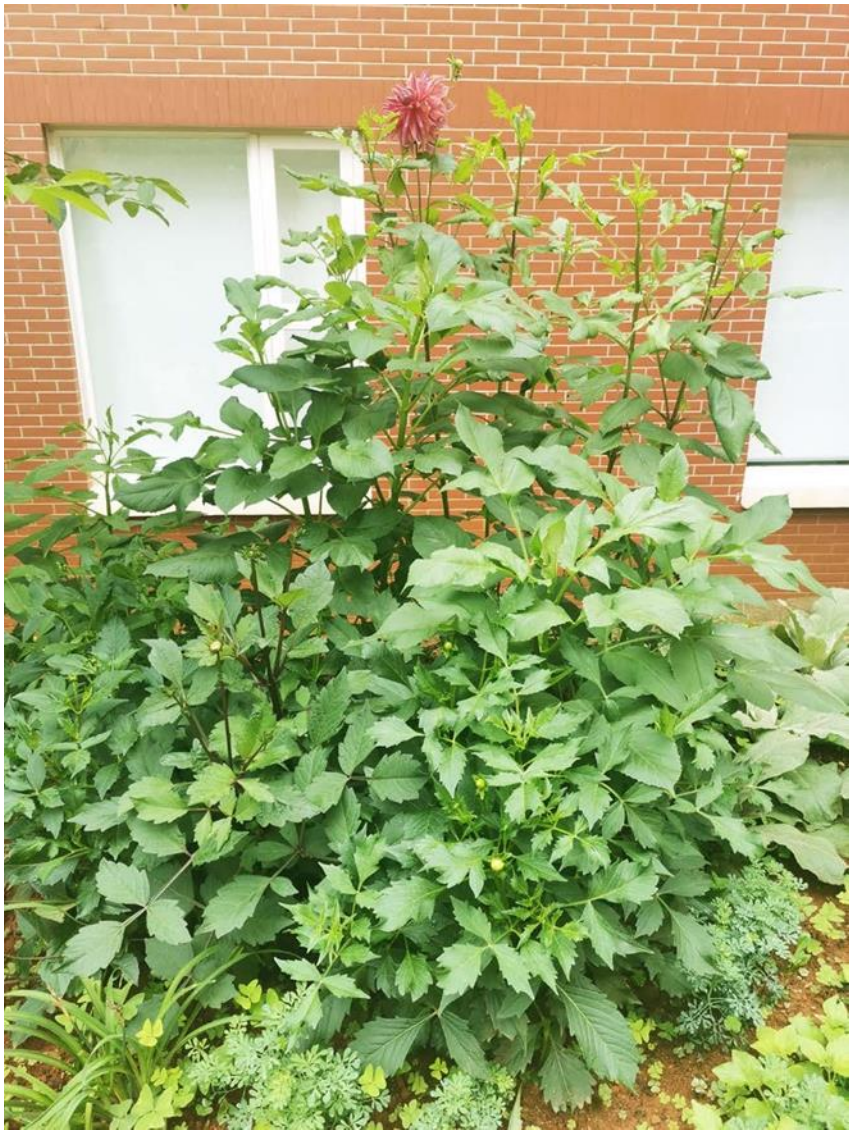

Supplementary Figure S1. *D. pinnata* plants grown outdoors showing their large biomasses.

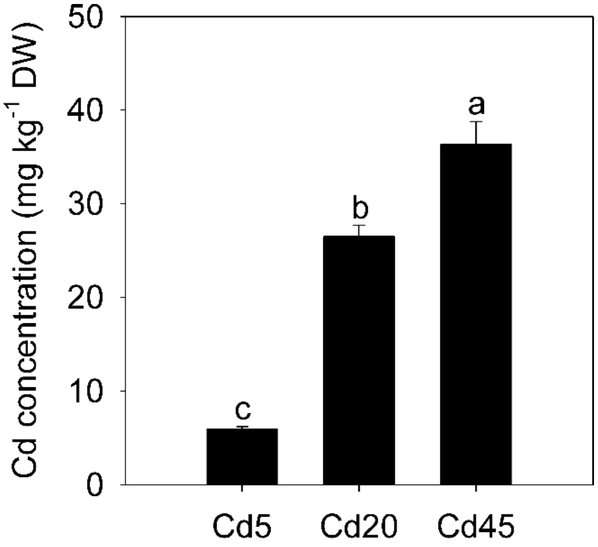

Supplementary Figure S2. Cd concentrations in shoots of *D. pinnata* in soils spiked with 5 (Cd5), 20 (Cd20), and 45 mg kg^{-1} Cd (Cd45). Data represent means ± standard deviations (n = 3). The bars labeled with different letters indicate significant differences at the $P < 0.05$ level among different groups according to Tukey's test. DW: dry weight.

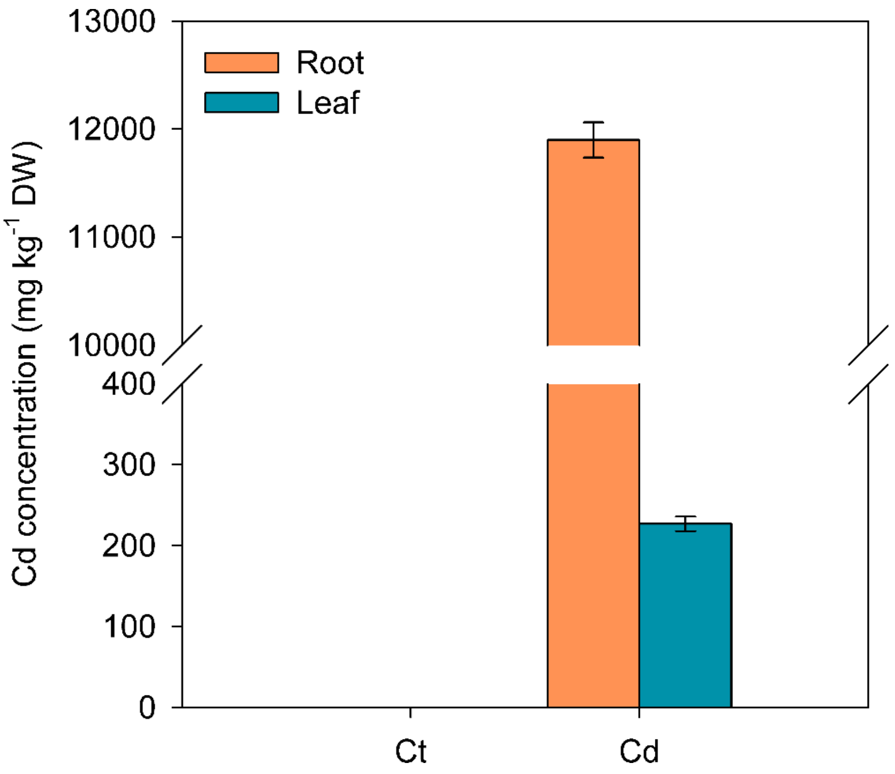

Supplementary Figure S3. Cd concentrations in roots and leaves of *D. pinnata* grown in control (Ct) and Cd treatment (50 mM, 48 h) conditions.

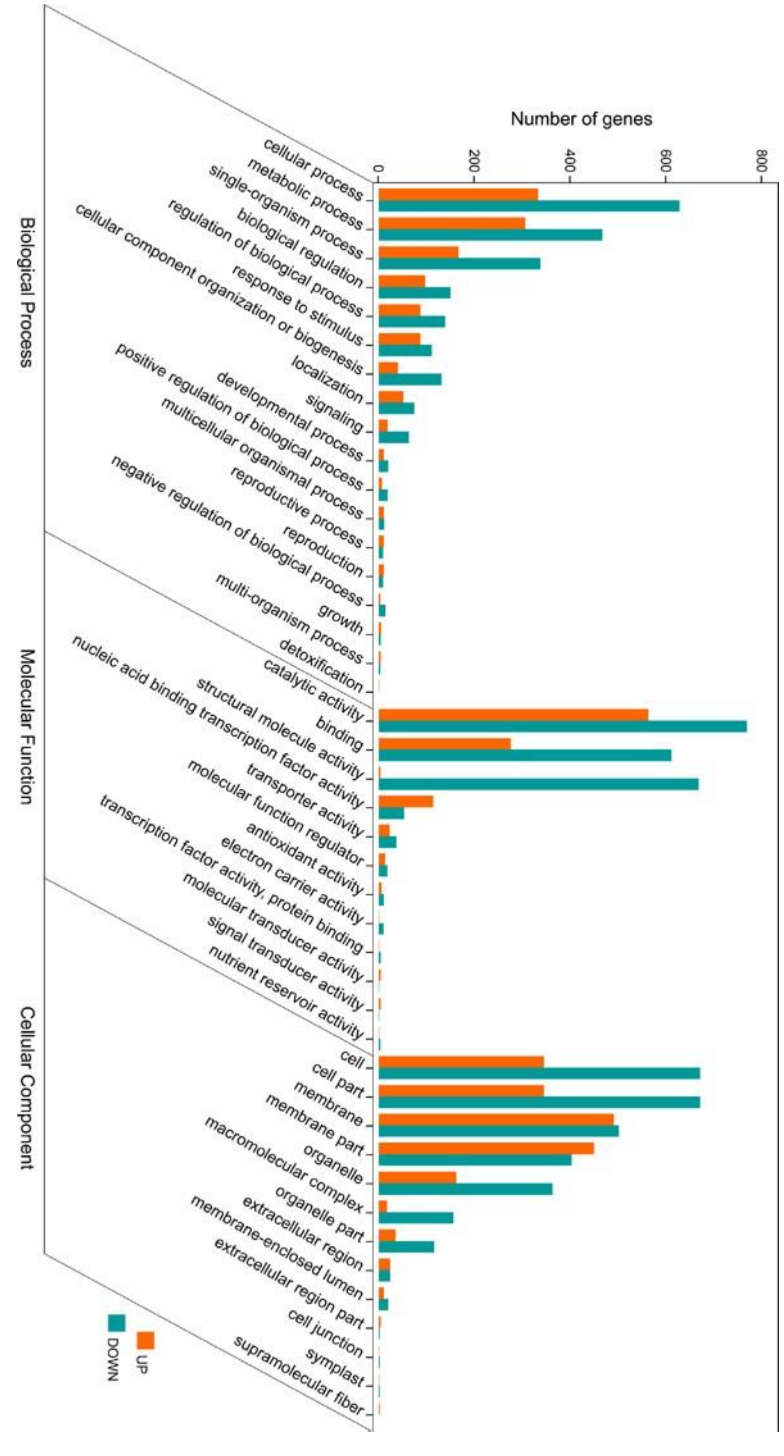

Supplementary Figure S4. GO enrichment analysis of differentially expressed genes in roots of *D. pinnata* grown in Cd treatment (50 mM, 48 h) condition compared with control condition.

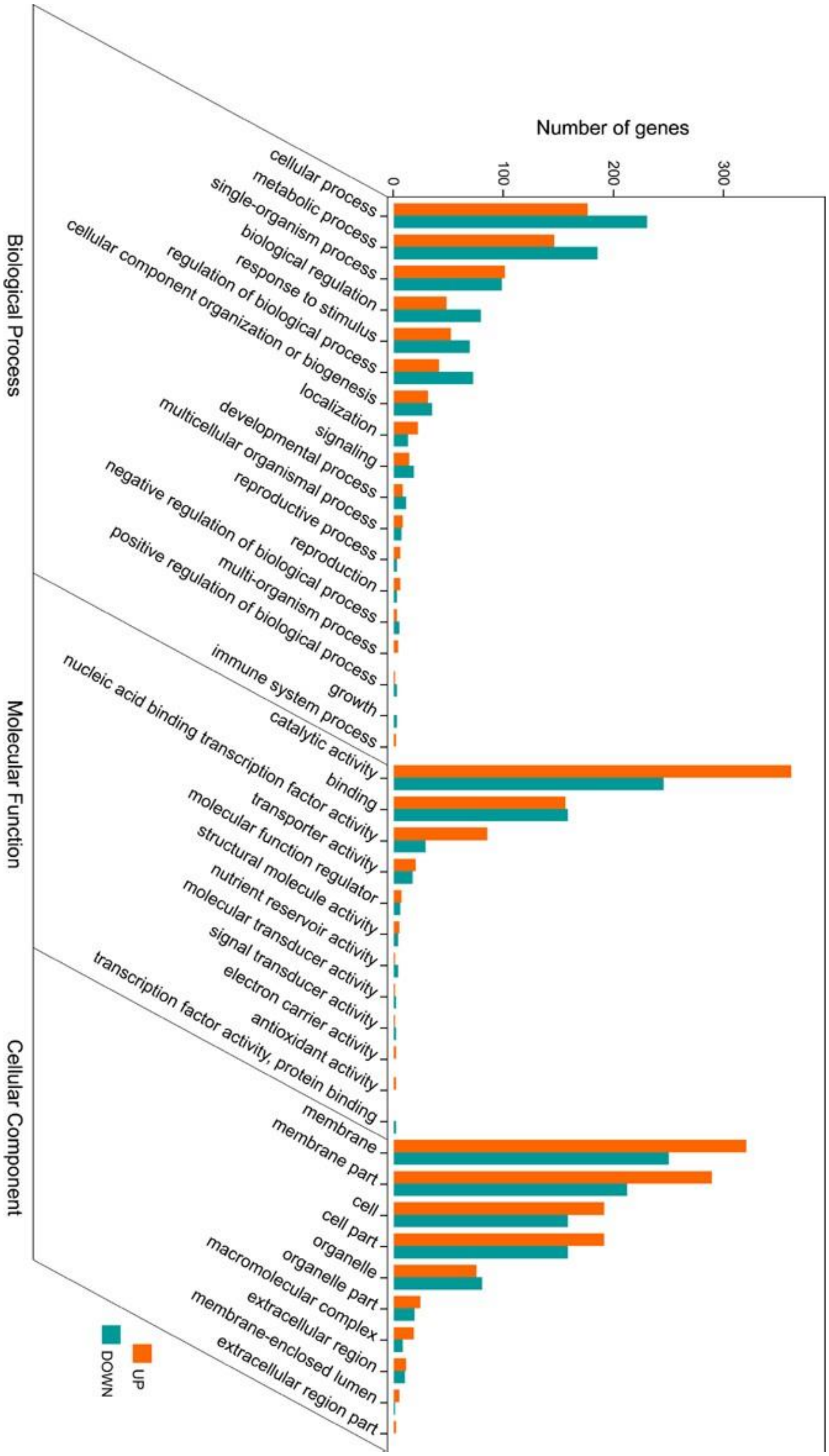

Supplementary Figure S5. GO enrichment analysis of differentially expressed genes in leaves of *D. pinnata* grown in Cd treatment (50 mM, 48 h) condition compared with control condition.

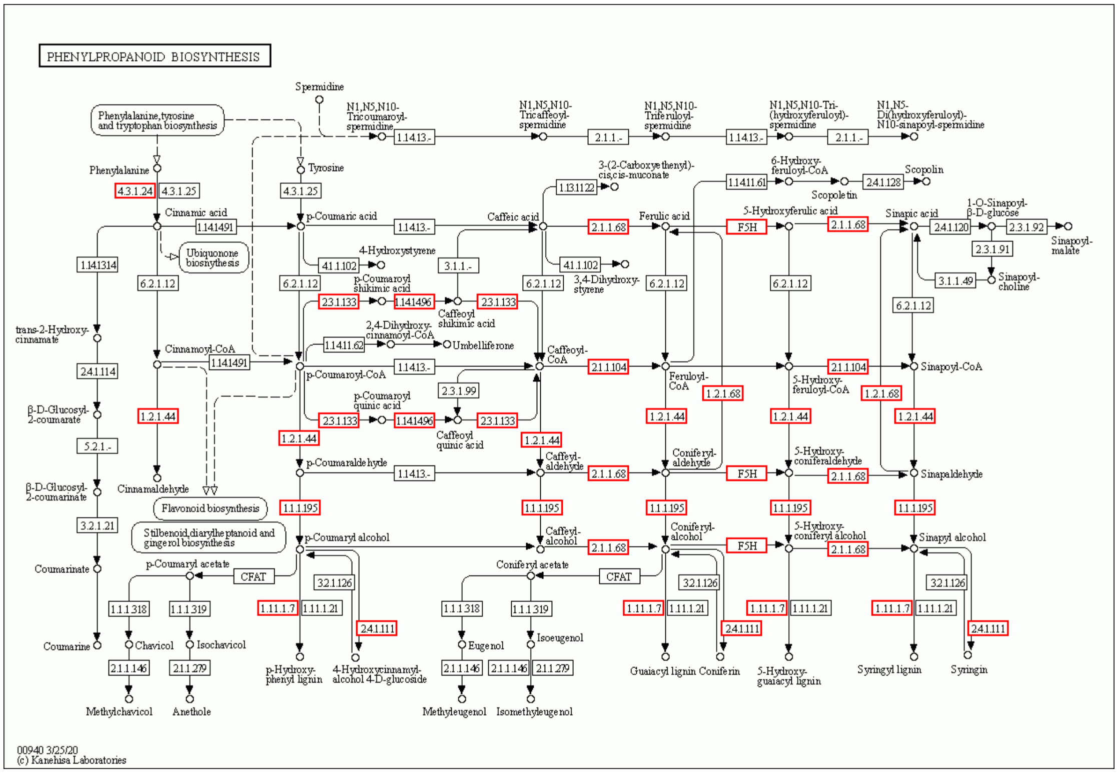

Supplementary Figure S6. Upregulated genes located on the phenylpropanoid biosynthesis pathway (red marks) in roots of *D. pinnata* grown in Cd treatment (50 mM, 48 h) condition compared with control condition.

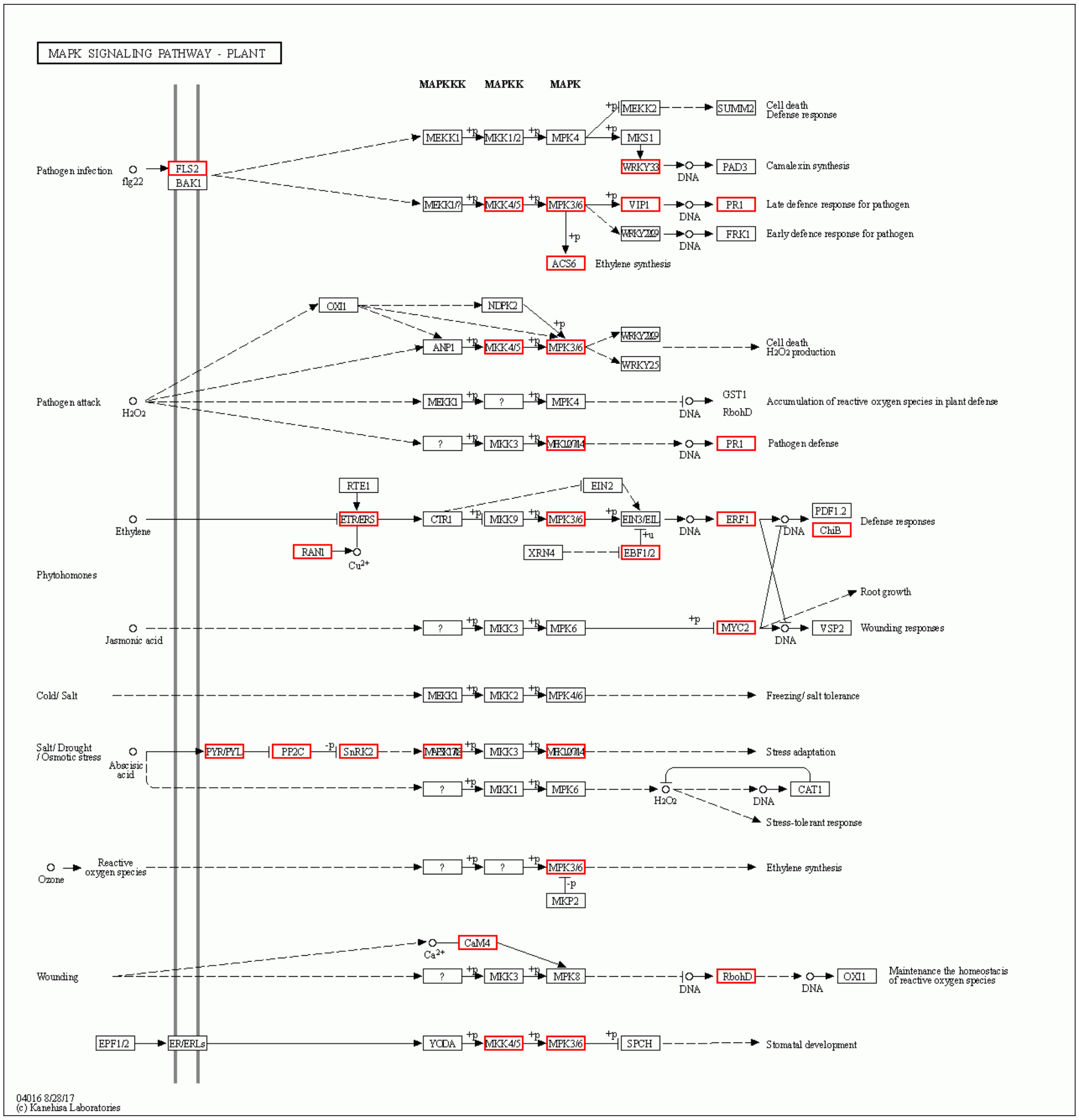

Supplementary Figure S7. Upregulated genes located on the MAPK signaling pathway (red marks) in roots of *D. pinnata* grown in Cd treatment (50 mM, 48 h) condition compared with control condition.

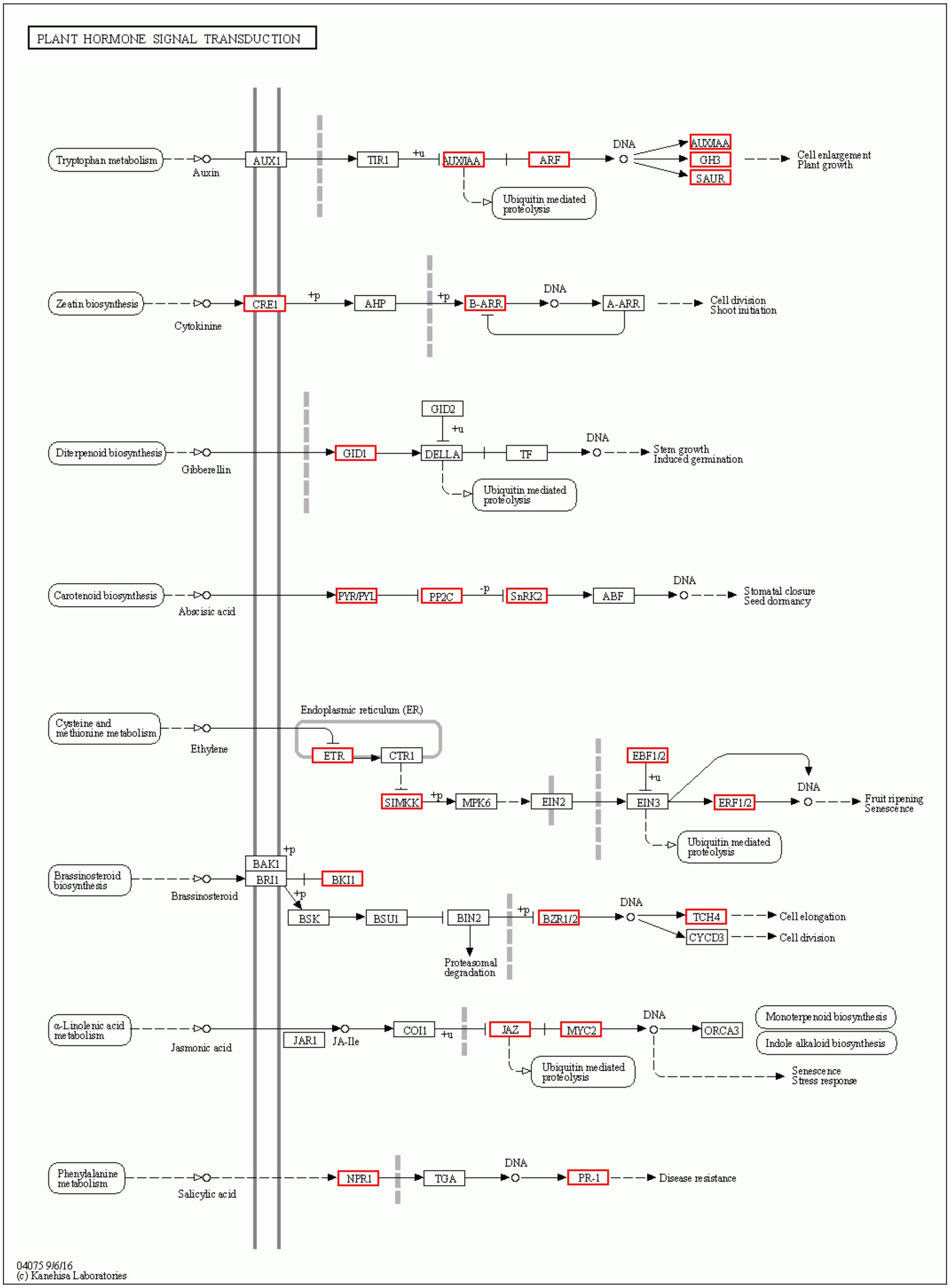

Supplementary Figure S8. Upregulated genes located on the plant hormone signal transduction pathway (red marks) in roots of *D. pinnata* grown in Cd treatment (50 mM, 48 h) condition compared with control condition.

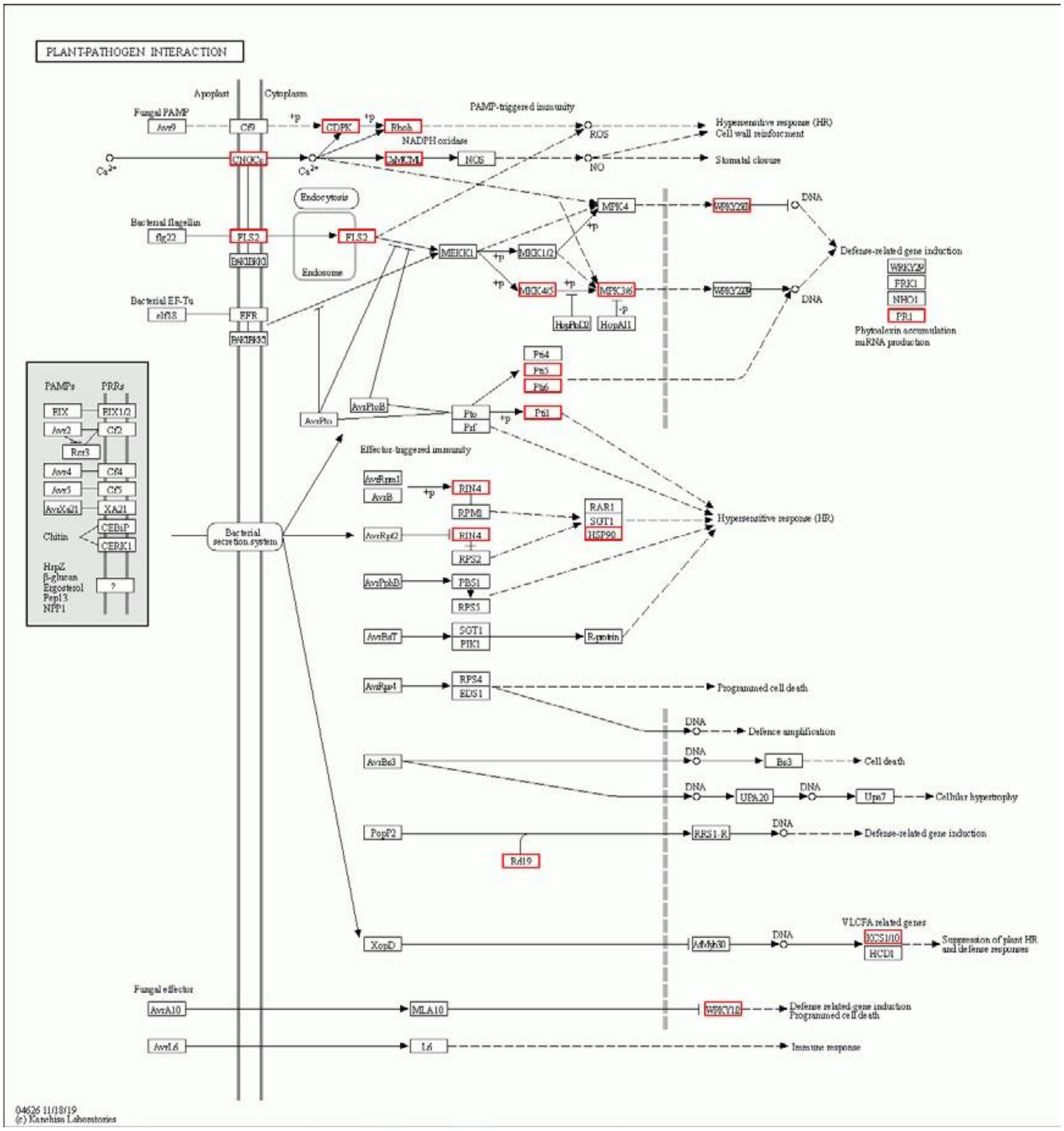

Supplementary Figure S9. Upregulated genes located on the plant-pathogen interaction pathway (red marks) in leaves of *D. pinnata* grown in Cd treatment (50 mM, 48 h) condition compared with control condition.

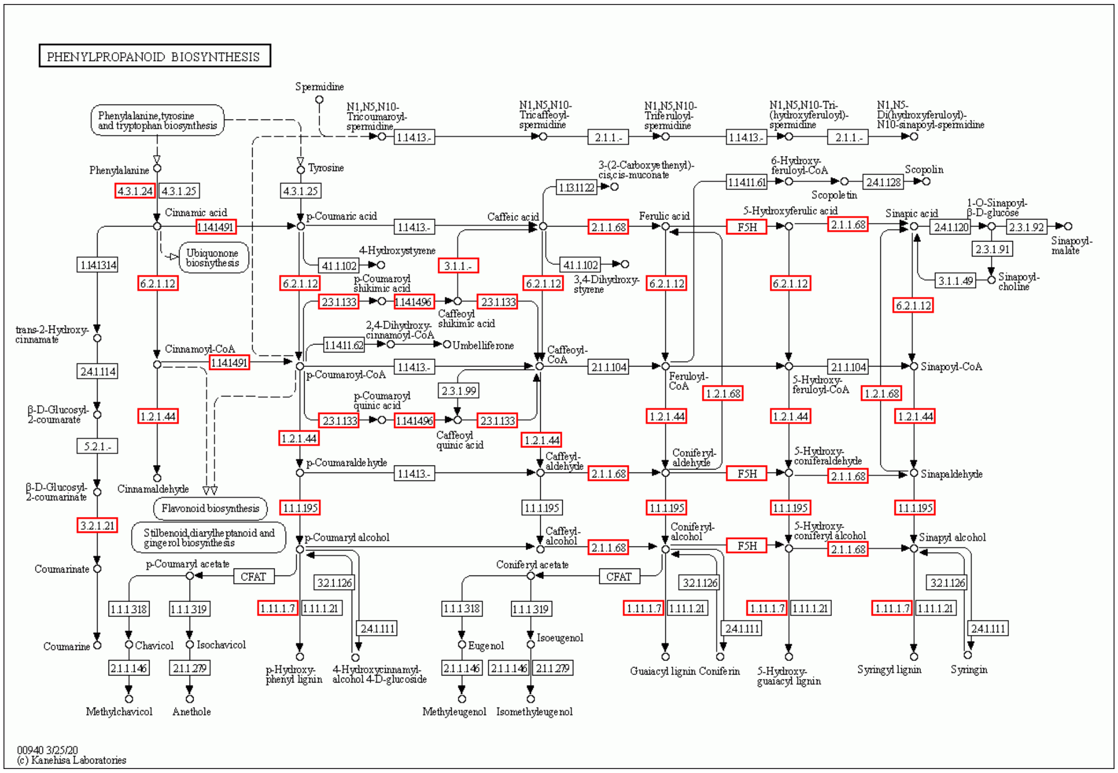

Supplementary Figure S10. Upregulated genes located on the phenylpropanoid biosynthesis pathway (red marks) in leaves of *D. pinnata* grown in Cd treatment (50 mM, 48 h) condition compared with control condition.

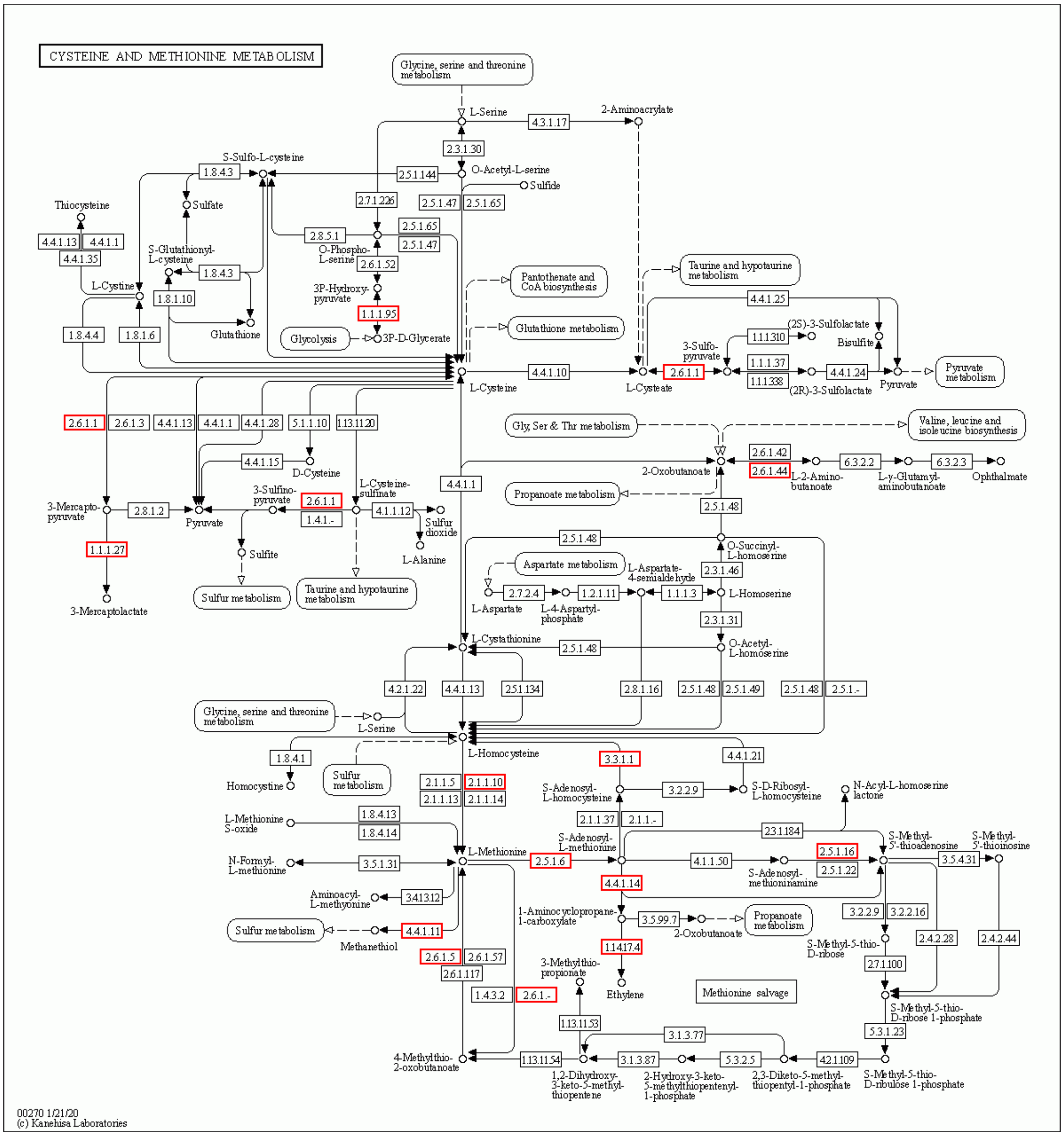

Supplementary Figure S11. Upregulated genes located on the cysteine and methionine metabolism pathway (red marks) in leaves of *D. pinnata* grown in Cd treatment (50 mM, 48 h) condition compared with control condition.
